# Effects of Slit Edge Notches on Mechanical Properties of 3D-Printed PA12 Nylon Kirigami Specimens

**DOI:** 10.3390/polym15143082

**Published:** 2023-07-18

**Authors:** Jing Shu, Junming Wang, Zheng Li, Kai-yu (Raymond) Tong

**Affiliations:** 1 Department of Biomedical Engineering, The Chinese University of Hong Kong, Hong Kong SAR 999077, China; 1155138492@link.cuhk.edu.hk (J.S.); 1155151867@link.cuhk.edu.hk (J.W.); 2 Department of Surgery, The Chinese University of Hong Kong, Hong Kong SAR 999077, China

**Keywords:** kirigami-inspired structures, kirigami slit notches, 3D printing, multi-jet fusion technique, wearable devices

## Abstract

Kirigami structures, a Japanese paper-cutting art form, has been widely adopted in engineering design, including robotics, biomedicine, energy harvesting, and sensing. This study investigated the effects of slit edge notches on the mechanical properties, particularly the tensile stiffness, of 3D-printed PA12 nylon kirigami specimens. Thirty-five samples were designed with various notch sizes and shapes and printed using a commercial 3D printer with multi-jet fusion (MJF) technique. Finite element analysis (FEA) was employed to determine the mechanical properties of the samples computationally. The results showed that the stiffness of the kirigami samples is positively correlated with the number of edges in the notch shape and quadratically negatively correlated with the notch area of the samples. The mathematical relationship between the stretching tensile stiffness of the samples and their notch area was established and explained from an energy perspective. The relationship established in this study can help fine-tune the stiffness of kirigami-inspired structures without altering the primary parameters of kirigami samples. With the rapid fabrication method (e.g., 3D printing technique), the kirigami samples with suitable mechanical properties can be potentially applied to planar springs for hinge structures or energy-absorbing/harvesting structures. These findings will provide valuable insights into the development and optimization of kirigami-inspired structures for various applications in the future.

## 1. Introduction

The term ‘kirigami’ originates from the Japanese words ‘kiri’ and ‘gami,’ which translate to ‘cut’ and ‘paper,’ respectively. [1]. In kirigami, paper sheets or thin wood are cut into specific patterns to create abstract or figurative forms, resulting in artwares [2]. In recent years, kirigami has emerged as a promising design paradigm in engineering, with its ability to enhance the stretching performance of materials by transferring stretching to bending (i.e., buffering-by-buckling) [3]. The resulting kirigami-inspired structures have found applications in various fields, including intelligent robotics [4,5,6,7,8], smart sensors [9,10,11,12,13,14,15,16], energy absorption structures [17,18,19], biomedical implants [20], deployable solar panels [21], and stretchable nanogenerators [22]. The use of kirigami in engineering designs reflects its potential to enable the creation of complex, multi-functional structures that can adapt to different environments and stimuli, with potential applications in a wide range of fields, including robotics, biomedicine, energy harvesting, and sensing. The incorporation of kirigami in engineering design is indicative of its capacity to facilitate the development of complex, multifaceted structures that can acclimate to varying environments and stimuli. The amalgamation of kirigami with advanced manufacturing techniques, such as 3D printing [23] and smart materials (4D printing [24]), further amplifies its capabilities.

Due to the significant potential for engineering design applications, researchers have recently explored the mechanical properties of kirigami-inspired structures. Similar to origami structures that fold the planar materials [25], kirigami-inspired structures are also capable of undergoing out-of-plane deformations. Isobe et al. [1] investigated the mechanical response of the Kent paper and examined the relationship between the geometrical parameters of cuts and the resulting mechanical behavior. Their findings revealed that the deformation of kirigami-inspired structures initiates with in-plane deformation, followed by out-of-plane deformation, resulting in a significant regime of stiffness reduction during the state transition. The transition between these two regions is determined by the interplay between in-plane and out-of-plane deformation energy [1]. Additionally, the thickness of the kirigami sample affects the range of in-plane deformation, which is positively correlated with the geometry thickness [1]. Han et al. [26] investigated the critical conditions of in-plane to out-of-plane deformation transitions of kirigami metallic glass with respect to the unit sizes of kirigami samples. Nakajima et al. [27] compared the mechanical properties of flexible kirigami samples (3D-printed with thermoplastic polyurethane (TPU) materials) with respect to different slit height values.

While there have been numerous studies on the effects of general geometry parameters of kirigami-inspired structures on their mechanical properties [13,28,29,30,31,32,33,34,35,36], only a few studies have investigated the effects of slit edge shape on the mechanical properties of kirigami samples. During stretching, stress is concentrated at the slit edges, potentially affecting the overall mechanical performance [37]. Research in [38,39,40] discussed the fatigue strength of samples with desired notches. With proper designs (e.g., round/circular-edge design), stress concentration can be significantly reduced [41]. In another study, Fujita et al. [31] compared the mechanical properties of cellulose nanopaper with normal edges and circular edges. Wang et al. [35] explored the stress distribution of kirigami samples with normal edges, circular edges, and rectangular edges. Chen et al. [28] fabricated several metallic glass kirigami samples with different round edge parameters and compared their stretchability. In this study, we present a systematic investigation of the effects of slit edge notches on the mechanical properties of kirigami specimens.

By leveraging 3D printing, or additive manufacturing, it is possible to fabricate kirigami-inspired structures with relative ease [19,22,27,42]; furthermore, their mechanical properties can be customized by selecting appropriate materials. The range of materials available for use encompasses both conventional and biocompatible polymers [43,44]. With the aid of 3D printing, or additive manufacturing, kirigami-inspired structures can be easily fabricated, and their mechanical properties can be tailored by selecting different materials. In this study, we utilized polyamide 12 (PA12) as the material for our kirigami samples. Polyamide, commonly known as nylon, has been widely used since the 1930s [45]. Its semi-crystalline structure provides well-balanced mechanical properties, with the crystalline regions contributing to its rigidity, yield strength, creep resistance, chemical resistance, and melt temperature, while the amorphous regions provide impact resistance, permeability, thermal expansion, and elastic properties [46]. In the study conducted by Hamzehei et al. (2023), it was demonstrated that the use of PA12 material in 3D printing of metamaterials resulted in remarkable recoverable behavior and exceptional energy absorption and dissipation properties [47].

**Figure 1:**
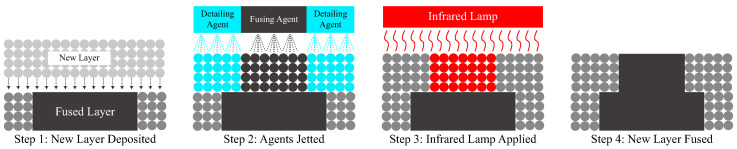
Sketches of the multi-jet fusion (MJF) printing process (modified based on [55]).

In recent years, a variety of additive manufacturing techniques have emerged and gained significant attention [48,49]. According to ISO/ASTM 52900 standard [50], these techniques can be categorized into seven distinct categories, namely binder jetting, direct energy deposition, material jetting, sheet lamination, VAT photopolymerization, material extrusion, and powder bed fusion (PBF). Fused filament fabrication (FFF) [51,52,53,54], one of the material jetting additive manufacturing methods, and PBFpowder bed fusion (PBF) [55,56,57,58,59] are common additive manufacturing techniques for polyamide. Selective laser sintering (SLS) and multi-jet fusion (MJF) are two well-established PBF processes widely used in the industry for the production of polymeric components [49]. PA12 is the most commonly used polymer in both PBF processes due to the wide temperature gap between the onset melting temperature and the onset crystallization temperature [55]. Compared with FFF and SLS methods that generate micro-gaps between layers [60], the MJF technique does not create porosities in the fabricated part [56], which increase the uniformity and weaken the anisotropy in the tensile strength [61]. The depiction of the MJF printing process is presented in Figure 1. Following the deposition of a new layer of material powders (step 1), the fusing agent is sprayed onto the target region, while the water-based detailing agent is sprayed onto the surrounding area (step 2) [55]. Upon exposure to infrared lamp radiation (step 3), the fusing agent absorbs heat and subsequently fuses the printing material powder. The detailing agent exhibits a temperature increase in the surrounding powders, resulting in improved details on the final printed part, as compared to SLS technology [38]. In our investigation, we leveraged this feature of MJF printing technology to enhance the preservation of details at the notches of kirigami samples. Therefore, we utilized the MJF 3D printing technique to fabricate the PA12 kirigami samples in our study. Moreover, MJF printing can provide more details on the printing part edge compared to SLS technology [38]. Therefore, in our study, we employed the MJF 3D printing technique to fabricate the PA12 kirigami samples.

Overall, this paper presents a comprehensive analysis and comparison of the effects of slit edge notches on the mechanical properties of kirigami specimens additively manufactured with nylon material. Overall, this paper provides a comprehensive comparison and analyzes the effects of slit edge notches on the mechanical properties of 3D-printed Nylon kirigami specimens. Both finite element analysis (FEA) and experiments were employed in the analysis. The design and fabrication of the kirigami samples are presented in Section 2. Section 3 focuses on the experimental setup. The experimental results and discussions are illustrated in Section 4, followed by the conclusion in Section 5.

## 2. Design and Fabrication of Kirigami Samples

**Figure 2:**
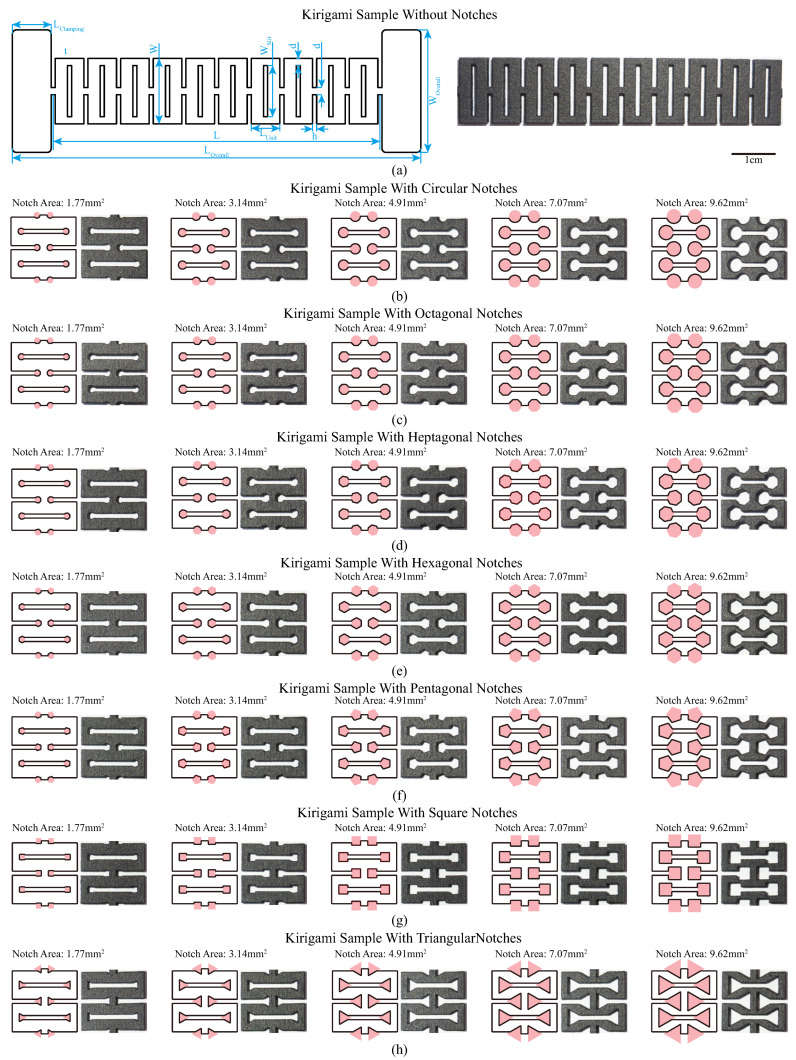
Kirigami samples with different slit edge notch sizes and shapes. Kirigami sample (**a**) without notches (a scale bar of 1 cm is presented); (**b**) circular notches; (**c**) octagonal notches; (**d**) heptagonal notches; (**e**) hexagonal notches; (**f**) pentagonal notches; (**g**) square notches and (**h**) triangular notches. Pink regions are used to express notches.

The kirigami samples with different slit edge notch sizes and shapes are shown in Figure 2. The original design of the kirigami pattern is presented in Figure 2a with dimensional parameters (presented in Table 1). The overall length $L_{Overall}$ and width $W_{Overall}$ of the sample are 100 mm and 30 mm, respectively. Two clamping regions are located on the two ends of the sample for fixing the sample in the tensile test. The kirigami patterns are placed in the middle, with a length *L* of 80 mm and width *W* of 16 mm. To ensure uniformities during stretching, ten periodic units are designed in one sample. The thickness *t* of the kirigami sample is set to 3 mm to eliminate the effect from printing tolerance in thickness. The slit height *h* of the kirigami sample is set to 1 mm to avoid the adherence of neighboring units. The slit weight $W_{Slit}$ is set to 12.5 mm. From [1,27], the kirigami sample’s stiffness $k_{1}$ during the first stage of the deformation (in-plane deformation) is as follows:

(1) $$k_{1}\propto E\cdot {\left(\frac{d}{W_{Slit}}\right)}^{3}\cdot t$$

where *E* is the Young’s modulus of the material. *d* represents the distance between two slits.

In this study, we analyze the influence of slit edge notches on the mechanical properties of 3D-printed kirigami samples. As highlighted in Section 1, the implementation of appropriate edge notch configurations can lead to a significant reduction in stress concentration [41]. For example, circular-shaped notches can form a U-shape at the edge of the slit, thereby delaying the yielding of structures. Additionally, this study also explores the potential of convex polygon-shaped notches, which feature obtuse angles in their interiors when the edge lengths are identical and the edge number is larger than four. This unique characteristic of convex polygon-shaped notches can also alleviate the stress concentration during stretching. Given the size of the kirigami samples and the fabrication resolutions, we designed kirigami-inspired samples by incorporating seven different notch shapes, namely: regular circle, octagon, heptagon, hexagon, pentagon, square, and triangle, located at the end of the slit. To alleviate stress concentration during stretching, we designed seven different notch shapes (regular circle, octagon, heptagon, hexagon, pentagon, square, and triangle) at the end of the slit. It has been established in prior research that the mechanical properties of kirigami samples are influenced by the notch sizes [28]. In the current study, we adjusted the notch sizes based on the percentage of the area covered by the notches. The diameters of the circular notches varied from 1.5 mm to 3.5 mm at 0.5 mm intervals, corresponding to 21.43% to 50% of the unit length $L_{unit}$, resulting in five different notch areas (1.77 ${\mathrm{mm}}^{2}$, 3.14 ${\mathrm{mm}}^{2}$, 4.91 ${\mathrm{mm}}^{2}$, 7.07 ${\mathrm{mm}}^{2}$, and 9.62 ${\mathrm{mm}}^{2}$). To ensure consistency, the notch areas with the six other shapes followed those of the circular-shaped notches. We varied the diameter of the circular notch from 1.5 mm to 3.5 mm at 0.5 mm intervals, resulting in five different notch areas (1.77 ${\mathrm{mm}}^{2}$, 3.14 ${\mathrm{mm}}^{2}$, 4.91 ${\mathrm{mm}}^{2}$, 7.07 ${\mathrm{mm}}^{2}$, and 9.62 ${\mathrm{mm}}^{2}$), which were then applied to the other six notch shapes. These notches are highlighted in pink shadows in Figure 2b–h. The kirigami patterns were designed using Onshape${}^{®}$ (PTC, Inc., Boston, MA, USA), a cloud CAD tool. During the design process, we first extruded the original design of the kirigami sample, and then built a new sketch on top of it. We drew the notch shapes on the slit ends with one of the convex polygon edges attached to the slit endpoint. Finally, we removed the notches with a depth equal to the thickness of the sample.

For convex polygons with identical edge lengths, the edge length $L_{edge}$ and the distance $d_{center}$ between their center and edges follow

(2) $$\begin{array}{ccc} d_{center} & = & \sqrt{\frac{A}{n\cdot \tan\left(\frac{\pi }{n}\right)}} \end{array}$$

(3) $$\begin{array}{ccc} L_{edge} & = & 2\cdot d_{center}\cdot \tan\left(\frac{\pi }{n}\right) \end{array}$$

where *A* is the area of the convex polygons and *n* is the number of edges (n≥3). From Equation (2), the distance $d_{center}$ correlates positively with the number of edges *n*. The edge length would be defined by notch areas according to Equation (2) and Equation (3). In Figure 2, the kirigami designs involve two independent variables: (1) notch size and (2) notch shape. Varying these two elements leads to changes in the position of the notch center, which is a dependent variable. As the edge numbers of the convex polygons in the notch shapes increase, the notch centers move inwards toward the longitudinal axis of symmetry of the kirigami sample. Similar effects are observed when the notch areas increase.

To fabricate the kirigami samples, we employed the multi-jet fusion (MJF) 3D printing technique, as mentioned in Section 1. The printing process was introduced in [38,55,58,62]. During MJF 3D printing, fusing and detailing agents are selectively sprayed on the surface of a powder layer of the printing materials. Then, IR lamps scan the powder bed; during this process, the material powders are fused by the energy absorbed by the fusing agents. The detailing agents prevent the fusing of surrounding material powders on the printing profile, resulting in better details when compared with the SLSselective laser sintering (SLS) 3D printing technique [55]. In this study, we printed the samples using an industrial MJF 3D printer (Jet Fusion 5200, HP, Palo Alto, CA, USA) with black PA 12 material (HP, USA). The samples were placed in an X-build orientation based on ISO/ASTM 52921 [55,63], i.e., the kirigami pattern was parallel to the build plate.

## 3. Experimental Setup

### 3.1. Experimental Setup for the Tensile Test of Kirigami-Inspired Samples

**Figure 3:**
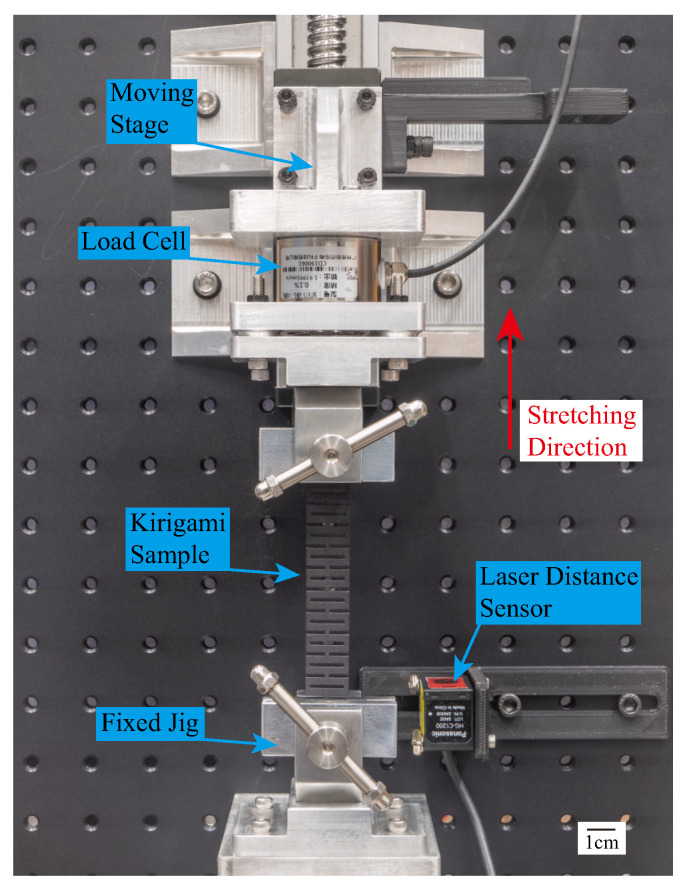
Experimental setup for kirigami-inspired sample stiffness testing.

The experimental setup for the tensile test is shown in Figure 3. To determine the mechanical properties of the kirigami-inspired samples, we built a customized tensile test machine. The samples were clamped by a pair of jigs, with one end fixed and the other end stretched by a moving stage driven by a screw slide. To avoid being affected by dynamic responses, the kirigami samples were stretched at a low speed of 5 mm/min (in accordance with the standard ASTM D638-14 [64], using the same testing speed as that used in quasi-static testing in previous studies [47,65]) until they broke. We used a load cell (SBT-673, SIMBATOUCH, Guangzhou, China) to measure the force generated during stretching, and a laser distance sensor (HG-C1200, Panasonic, Kadoma, Osaka, Japan) to measure the displacement of the screw slide. The force and displacement signals were recorded by a PC with a connected data acquisition (DAQ) device (USB6210, NI, Austin, TX, USA), and the DAQ frequency was set to 1000 Hz.

### 3.2. Experimental Setup for Finite Element Analysis (FEA)

**Figure 4:**
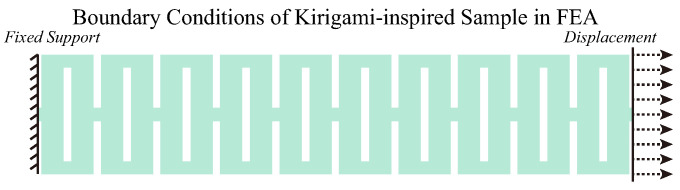
Illustration of FEA settings. The boundary conditions (fixed support and displacement) are indicated.

Finite element analysis (FEA) was employed during the design process of the kirigami samples to compare the differences in their mechanical properties. This allowed us to determine the characteristics of the samples without performing experiments. The FEA processes were performed on Ansys Workbench R15.0 (Ansys, Inc., Canonsburg, PA, USA). A static structural analysis was conducted, whereby the samples were tested in a quasi-static environment without the provision of dynamic information, such as speed and kinetic energy, among others. The mechanical properties of PA12 nylon are listed in Table 2 [59,66,67]. The value of Young’s modulus *E* was selected to be 1128 MPa according to the printing orientation of the kirigami samples [67]. The ‘multilinear isotropic hardening’ block was selected when setting the engineering data of the material, and the true stress and true plastic strain data [59] were filled into ‘uniaxial plastic strain test data.’ The screenshot of material settings is presented in Figure A2. Boundary conditions in FEA are shown in Figure 4. In the FEA, extruded cuts were made to create clamping regions for the tensile test. One end of each sample was assigned with fixed support, while the other end was stretched. The deformation value for each kirigami sample was identical to the elongation at the break in the tensile test presented in Section 3.1. The mesh settings in the FEA are presented in Table 3. The screenshot of mesh setups in the Ansys Workbench user interface is shown in Figure A3. The ‘automatic method’ was implemented during the meshing process, which involved sweeping the kirigami samples first for quadrilateral element generation. If sweeping was not feasible, the meshing algorithm generated four-node tetrahedral elements using the patch-conforming method. In our study, the kirigami samples were successfully swept, resulting in the establishment of quadrilateral elements and more accurate simulations. After FEA, the resistive force and stress versus the deformation of kirigami-inspired samples were determined.

In the FEA, extruded cuts were made to create clamping regions for the tensile test. One end of the samples was assigned with fixed support and another end was stretched. The deformation value for each kirigami sample was identical to the elongation at the break in the tensile test presented in Section 3.1. After FEA, the resistive force and stress versus the deformation of kirigami-inspired samples were determined.

## 4. Experimental Results and Discussion

**Figure 5:**
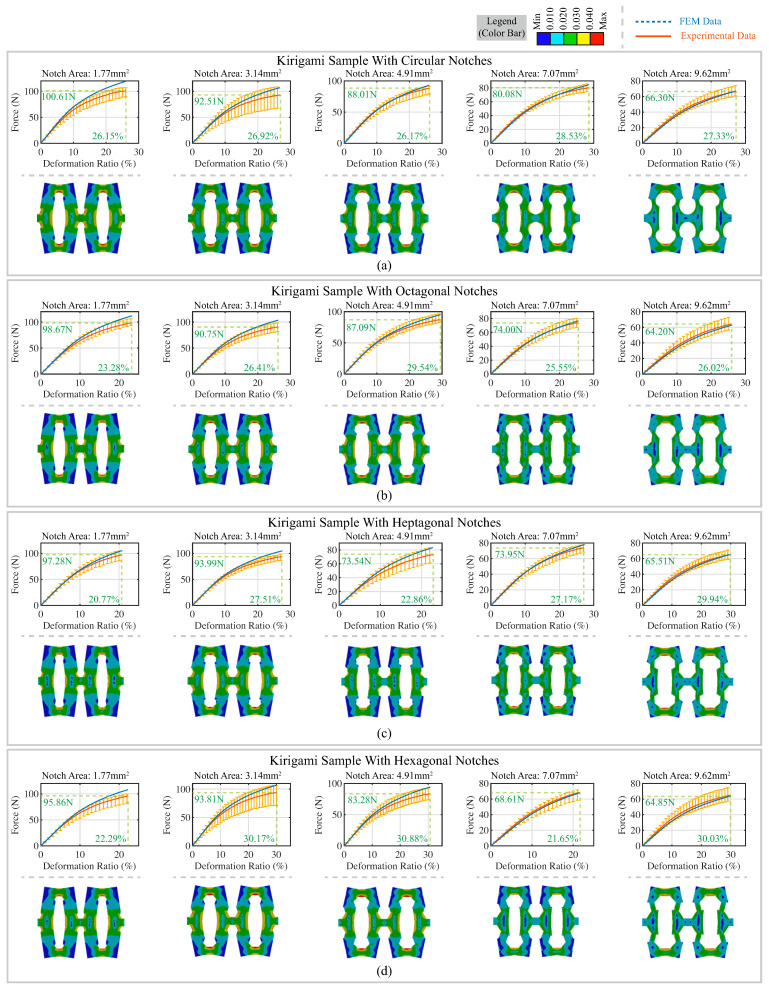

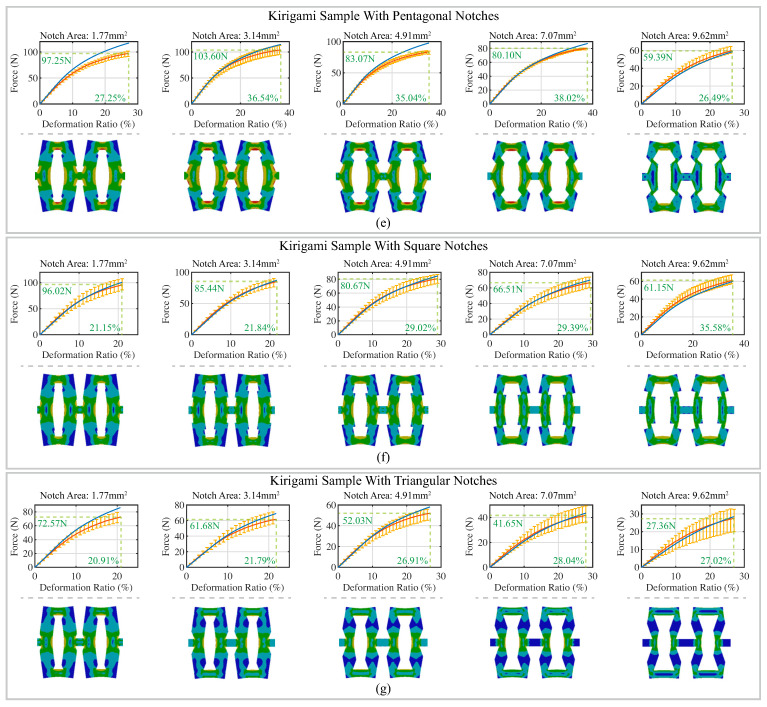
Tensile experimental results and FEA testing results of kirigami samples with (**a**) circular notches; (**b**) octagonal notches; (**c**) heptagonal notches; (**d**) hexagonal notches. Tensile experimental results and FEA testing results of kirigami samples with (**e**) pentagonal notches; (**f**) square notches; (**g**) triangular notches. The experimental data and FEA testing data are depicted using a solid red line and a dashed blue line, respectively, in the plot. A snapshot of the kirigami samples in FEA testing with strain distribution is presented in the bottom subfigure.

The experimental results of the tensile tests and FEA results are presented in Figure 5. The top part of each subfigure shows the relationship between the force and deformation ratio of the kirigami samples with different notches. Four tensile tests were conducted for each kirigami sample to avoid occasional anomalies, and the average force values were calculated and plotted with red solid lines. The error bars shown with yellow vertical lines represent the boundary values of experimental data. The FEA simulation data are presented with blue dashed lines.

**Figure A1:**
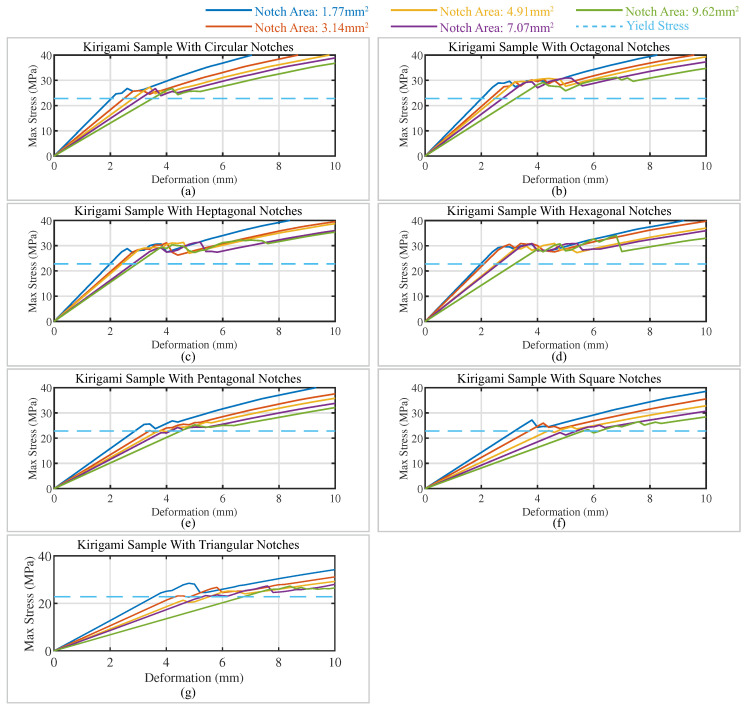
Maximum stress on kirigami-inspired samples with (**a**) circular notches; (**b**) octagonal notches; (**c**) heptagonal notches; (**d**) hexagonal notches; (**e**) pentagonal notches; (**f**) square notches; and (**g**) triangular notches during stretching.

**Figure A2:**
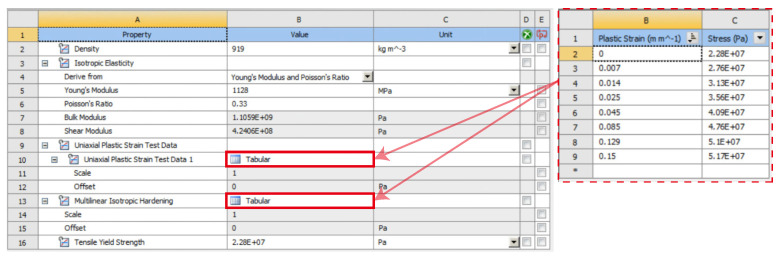
Screenshot of setup of material properties in finite element analysis testing.

**Figure A3:**
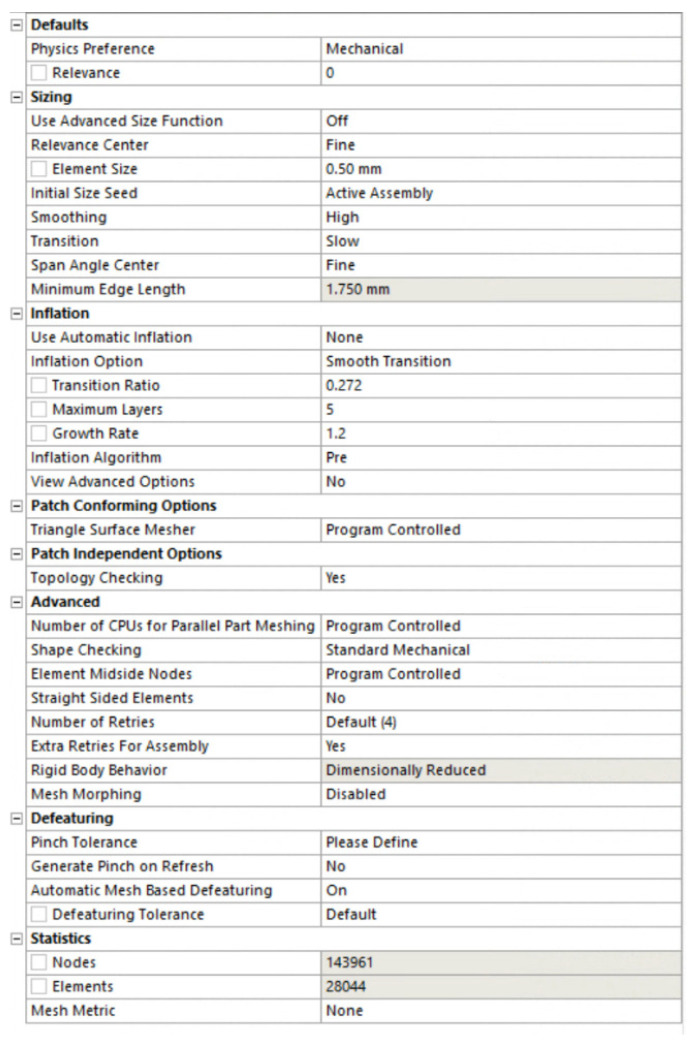
Screenshot of mesh settings in finite element analysis testing.

To better illustrate the deformation behavior of the kirigami structures, the FEA results were captured at the largest deformation point and the strain distribution is plotted in the bottom subfigure. The high strain regions were found to be concentrated near the notches. As the notch area increased, the strain values in the regions near the notches decreased. For example, the mass-tone of the region surrounding the central notches shifted from a green color (0.02 ≤ϵ≤ 0.03) to blue (strain value ϵ≤ 0.010), which postponed the onset of yielding during stretching. These observations were corroborated by the results of finite element analysis presented in Figure A1 (Appendix A), where the maximum stress of individual elements during stretching was plotted for each kirigami sample. The yielding point of each sample was calculated, and the corresponding yielding deformations are presented in Table A1 (Appendix A). For each notch shape, the yielding deformation increased with the notch area.

For kirigami samples with identical notch shapes, the fracture force (i.e., the force generated at the breaking point) exhibited a negative correlation with the notch area. From Figure 5, the experimental data and FEA simulation results have similar trends. However, as the deformation ratio increased, the gap between the experimental data and the FEA simulation results widened.

**Figure 6:**
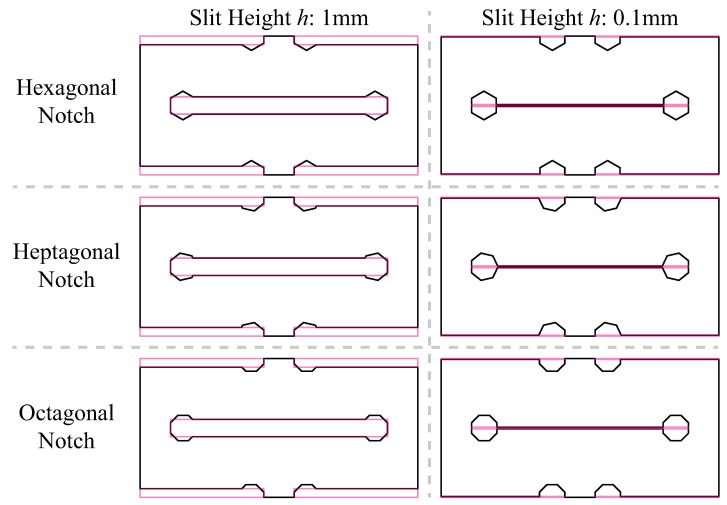
Enlarged sketch when the slit height of kirigami pattern reduced from 1 mm to 0.1 mm.

**Figure A4:**
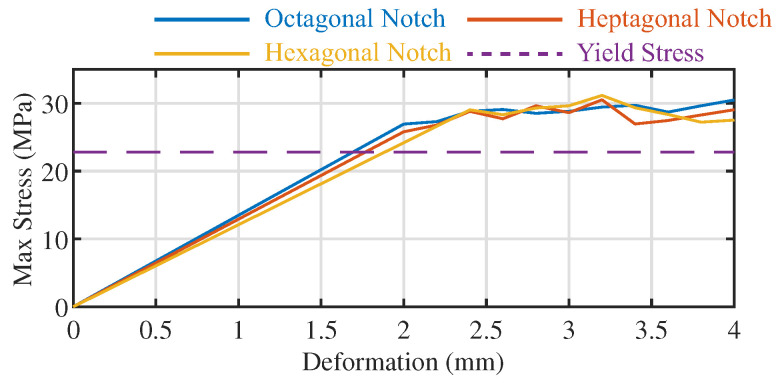
Maximum stress on slit height-reduced kirigami-inspired samples with octagonal, heptagonal, and hexagonal notches. The slit height of kirigami samples reduced from 1 mm to 0.1 mm.

In order to contrast the dissimilarities between experimental and finite element analysis data, the stiffness of kirigami samples exhibiting notches of varying dimensions was evaluated using the following relationship:

(4) $$K=\frac{F_{\lambda_{0}}}{\Delta L_{\lambda_{0}}},$$

where *K* represents the stiffness of the kirigami samples, while $F_{\lambda_{0}}$ and $\Delta L_{\lambda_{0}}$ correspond to the force and displacement values, respectively, at the yielding point during stretching. The results of the calculations, including errors and error percentages, are presented in Table 4. The error percentage was calculated based on

(5) $$Error\;Percentage\;\left(\%\right)=\frac{K_{Experiment}-K_{FEA}}{K_{FEA}}\times 100\%,$$

where $K_{Experiment}$ and $K_{FEA}$ are the stiffness calculated by experimental and FEA data, respectively. As presented in Table 4, it can be observed that the stiffness of the kirigami sample generally increases with an increase in the number of edges in the notch area, while maintaining a constant notch area (wherein the circle is considered a polygon with an infinite number of edges). Based on Equation (2), the distance *d* between the center of the polygon and its edges increases with the number of edges. Therefore, a positive correlation exists between the stiffness of the kirigami sample and the distance *d*. However, an unexpected trend is observed in the stiffness of the kirigami samples with a notch area of 1.77 ${\mathrm{mm}}^{2}$, as both experimental and computational results reveal a relationship where $K_{\mathrm{Hexagon}}<K_{\mathrm{Octagon}}<K_{\mathrm{Heptagon}}$. This trend does not follow the positive correlation between the stiffness of the sample and the number of notch edges. This inconsistency can potentially be attributed to the value of the kirigami slit height *h* (refer to Figure 2). To avoid conglutination between the kirigami units during 3D printing, the value of *h* was set to 1 mm, which is relatively significant when compared to the notches. In Figure 6, the sketches of the kirigami unit are enlarged to depict the relative location of kirigami slits. In order to mitigate the impact of kirigami slits on the stretching stiffness, we conducted an updated set of FEA tests on kirigami samples with reduced slit heights. These samples featured octagonal, heptagonal, and hexagonal notches with areas measuring 1.77 ${\mathrm{mm}}^{2}$. The sketches of the kirigami sample units with reduced slit heights to 0.1 mm are displayed on the right-hand side of Figure 6. The maximum stress sustained by individual elements during the stretching of these kirigami samples is presented in Figure A4. The yield deformation and stiffness estimated from FEA are tabulated in Table A3. It is observed that, as the slit height is reduced to a negligible level with respect to the notch area, the stiffness of the kirigami sample is found to be positively correlated with the number of notch edges, i.e., $K_{\mathrm{Hexagon}}<K_{\mathrm{Heptagon}}<K_{\mathrm{Octagon}}$. It is evident that as the number of edges in the notch area of the kirigami sample increases, the stiffness of the sample also increases, while keeping the notch area constant (note that the circle is treated as a polygon with an infinite number of edges). Based on Equation (2), the distance between the center of the polygon and its edges increases with the number of edges, resulting in a positive correlation between the stiffness of the kirigami sample and the distance *d*. This relationship can be explained from an energy perspective.

**Figure 7:**
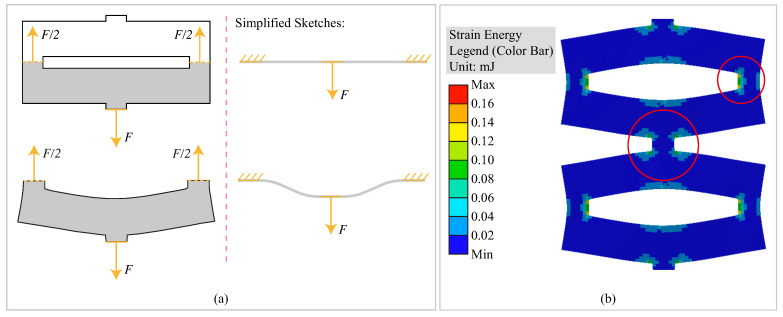
Deflection of the kirigami unit during stretching. (**a**) The sketches represent the states of the kirigami sample beams before and after stretching. The state before and after the stretching is shown in the upper part and bottom parts of the figure, respectively. The beam is shaded in gray. The deflection of the center part in the simplified sketches is exaggerated. (**b**) The strain energy distribution of the kirigami sample without notches when the deformation is 20 mm. Legends are presented on the left side of the figure. The element size was set to 0.25 mm to present more details. The high strain energy concentrated regions are highlighted using the red circle.

This relationship can be elucidated from an energy perspective. When considering a single unit of kirigami samples, it can be regarded as two combined beams, as depicted in Figure 7a, where the shaded gray portion represents the beam. The beam is subjected to a tensile force *F* applied to the center and two ends of the beam, which induces bending. The bending of the kirigami unit’s beam follows the theory of the three-point beam bending test, and a simplified illustration is presented on the right-hand side of Figure 7a, where the deflection of the center part is exaggerated. The location of the notches is stretched based on the three-point beam bending theory. A new finite element analysis was conducted on the original kirigami samples without notches, using a deformation of 20 mm and a finer element size of 0.25 mm to provide enhanced resolution. The strain energy of each element was calculated, and the resulting distribution is presented in Figure 7b. The figure illustrates that the high-strain energy regions are primarily concentrated at the ends of the cutting edges (highlighted in red circles). The introduction of notches to the sample results in the removal of the high-strain energy region and its replacement with a relatively lower-strain energy region surrounding the notch. For the selected notch shape, since the area is identical, a smaller value of the distance $d_{center}$ (i.e., closer to the cutting edge length) results in a removed area with denser strain energy. This generates less strain energy during the deformation of the entire sample and subsequently lowers its stretching stiffness, thus explaining the observed negative correlation between the stiffness of the kirigami samples, the notch size, and the number of edges in the notch shape. The finite element analysis results for the strain energy of kirigami samples are presented in Table A2 (Appendix A). It was observed that the strain energy of kirigami samples increased with the number of edges, which indicated a greater stiffness of the samples.

Through the use of FEA and the error percentage analysis, we were able to estimate the stiffness of kirigami samples with specific notch areas without the need for experimental testing. The absolute error value presented in Table 4 initially decreases and then increases as the notch area increases. Consequently, the FEA methods employed in this study demonstrate better estimation performance when the notch area falls within the range of 3.14 to 4.91 ${\mathrm{mm}}^{2}$.

**Figure 8:**
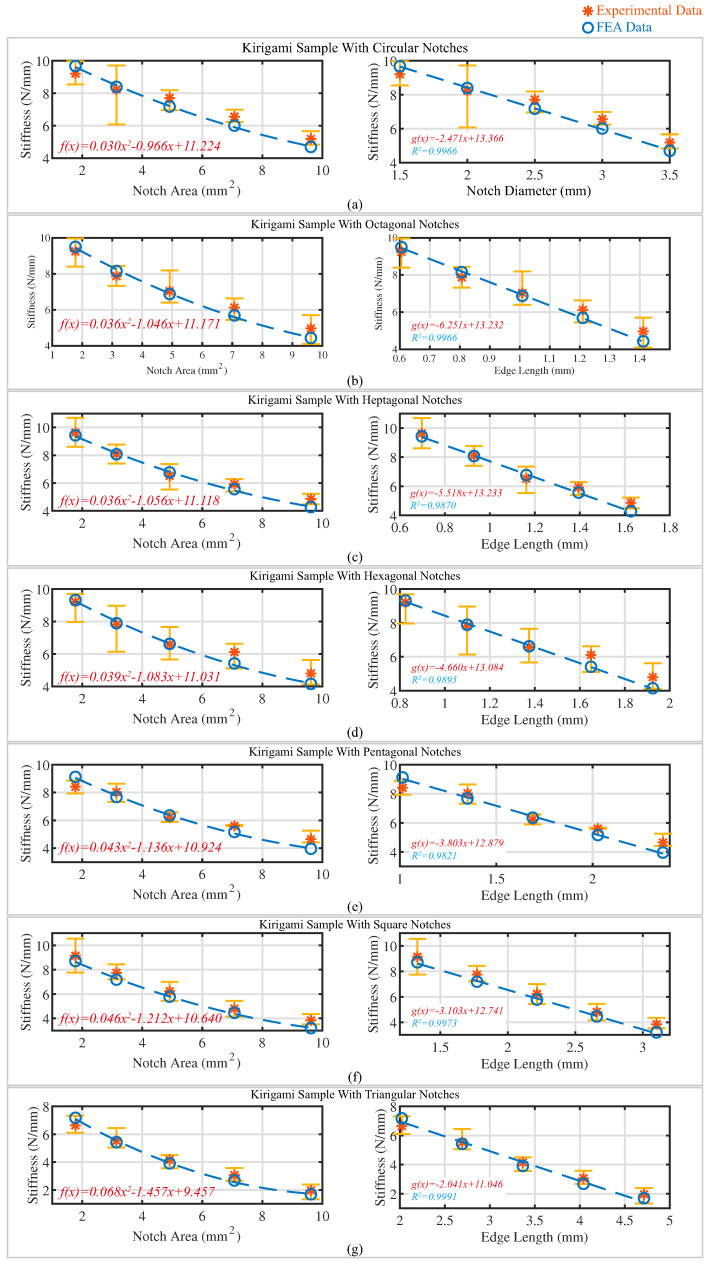
The relationship between the stiffness of the kirigami sample and two parameters: (1) the notch area and (2) the edge length (diameter for round notches) for kirigami samples with (**a**) circular notches; (**b**) octagonal notches; (**c**) heptagonal notches; (**d**) hexagonal notches; (**e**) pentagonal notches; (**f**) square notches and (**g**) triangular notches. The computational mathematical relationship and coefficient of determination ($R^{2}$) (for the relationship between stiffness and edge length) are indicated.

Figure 8 depicts the relationship between the stiffness of the kirigami sample and two parameters: (1) the notch area and (2) the edge length (diameter for round notches). Each subfigure showcases the stiffness values plotted against the respective parameter, on the left and right sides, respectively. The stiffness values are obtained using the finite element analysis and experimentally measured data, represented by blue circles and red stars, respectively, with error bars indicating the range of values. Following the approach utilized in [27], we employed linear regression analysis to fit the stiffness values estimated through FEA for different notch shapes, with varying edge lengths (diameters for circular notches). The fitted relationships are represented using blue dashed lines shown in the subfigures on the right-hand side of Figure 8. The coefficient of determination ($R^{2}$) was calculated for kirigami samples with different notch shapes, and the value of $R^{2}$ was found to be close to 1, indicating a linear dependence of the kirigami sample’s stiffness on the edge length of the notches (diameter for round notches) mathematically. Moreover, we performed quadratic regression analysis for the FEA-estimated stiffness of samples and their corresponding notch area, represented as a blue dashed line plotted on the left-hand side of Figure 8. The mathematical relationships are presented in the lower left corners of each subfigure. Similar to the relationship between the stiffness of samples and the number of edges of notches, this observation can also be explained using the aforementioned energy theory. As the notch area increases, more regions of high-strain energy are removed, leading to a decrease in the strain energy during stretching, ultimately causing a reduction in the stiffness of the specimens. Our findings reveal a linear dependence of the kirigami sample’s stiffness on the edge length of notches (diameter for round notches) and a quadratic dependence on the area of the notch. This is consistent with the notion that the area and edge lengths of the notches are in a quadratic relationship. The mathematical relationships between the kirigami sample stiffness and the notch area/edge length are presented in the lower left corners of each subfigure, in red, through the mathematical fitting.

The relationship established in this study can be applied to the design and fabrication of wearable devices for human assistance. The mechanical properties of kirigami samples can be fine-tuned by applying different slit notches and changing the area, presenting a new approach for property tuning. Potential applications include planar springs for hinge structures (similar to our previous research in [68]) or energy-absorbing structures. The kirigami samples in this study were composed of ten repetitive units. In specific applications, the numbers and sizes of the kirigami units can be adjusted to suit the desired requirements.

However, the fabrication of kirigami specimens via 3D printing presents potential challenges and limitations. For instance, the range of available materials for 3D printing remains restricted, and certain specialized materials, such as metallic glass kirigami samples mentioned in the introduction, are currently impractical to produce via 3D printing. Additionally, due to the mechanics of 3D printing, the details of printed kirigami samples may not be as precise as those fabricated using conventional methods such as mold casting and CNC machining, which may have implications for the mechanical properties of the stress concentration regions, particularly the notches, in kirigami samples. Thus, the practical application of the findings presented in this study requires careful consideration of trade-offs. The notch sizes of kirigami samples must be sufficiently large to ensure accurate fabrication, while the base material must be chosen judiciously to optimize the overall mechanical performance of the samples.

## 5. Conclusions

This study investigated the effects of edge notches on the mechanical properties, particularly the stiffness during stretching, of 3D-printed PA12 nylon kirigami specimens. A total of 35 types of kirigami samples with different notch sizes and shapes were designed and fabricated using commercial 3D printers with the MJF printing technique. Force-deformation curves were generated for the samples, and the uniaxial tensile stiffness was determined by both experimental testing and finite element analysis (FEA). The acceptable error between experimental data and FEA results enabled the computation of mechanical properties of kirigami samples with unknown parameters. It was observed that the stiffness of the kirigami samples is positively correlated with the number of edges in the notch shape and quadratically negatively correlated with the notch area of the samples. The mathematical relationship between the stretching stiffness of the samples and their notch area was established, and this phenomenon was explained from an energy perspective. FEA was conducted to determine the strain energy distribution of kirigami samples during stretching, and the strain energy was computed accordingly. This paper proposes an alternative approach to modifying the mechanical properties of kirigami samples, specifically their tensile stiffness. Rather than altering the primary geometry parameters, the properties can be precisely adjusted by introducing notches of various geometries and sizes at the end of the slits. While 3D printing is demonstrated to be a facile method to fabricate kirigami samples with diverse geometries, the practical significance of the proposed approach is underscored by the potential to incorporate notches on conventionally fabricated kirigami samples, such as casted samples, to achieve desirable mechanical properties. This present study provides significant contributions to the development of kirigami-based devices, offering valuable insights into various fields, such as soft robotics, biomedical engineering, and flexible electronics. Further research is warranted to evaluate the feasibility and efficacy of kirigami-based devices in practical applications, and to assess the influence of various factors, such as material properties, geometric parameters (including the interplay between slit notches and slit height), and fabrication techniques, on the mechanical behavior of kirigami structures.

The relationship established in this study can be applied to the design and fabrication of wearable devices for human assistance. The mechanical properties of kirigami samples can be fine-tuned by applying different slit notches and changing their area, presenting a new approach for property tuning. Potential applications include planar springs for hinge structures (similar to our previous research in [68]) or energy-absorbing structures. The kirigami samples in this study were composed of ten repetitive units. In specific applications, the numbers and sizes of kirigami units can be adjusted to suit the desired requirements. The present study provides significant contributions to the development of kirigami-based devices, offering valuable insights into various fields, such as soft robotics, biomedical engineering, and flexible electronics. Future research can investigate the feasibility and performance of kirigami-based devices in practical applications, as well as the impact of different materials, geometries, and fabrication techniques on the mechanical properties of kirigami structures.

## Figures and Tables

**Table 1:** Dimensional parameters for kirigami-inspired testing samples.

Parameter	Description	Value (mm)
*W*	Width of Kirigami Pattern	16
$W_{Slit}$	Slit Width	12.5
$W_{Overall}$	Overall Width of Sample	30
*L*	Length of Kirigami Pattern	80
$L_{Overall}$	Overall Length of Sample	100
$L_{Clamping}$	Length of Clamping Region	9.5
$L_{Unit}$	Length of Single Unit	7
*d*	Distance Between Two Slits /Slits and Sample Edge	1.75
*h*	Kirigami Slit Height	1
*t*	Thickness of Sample	3

**Table 2:** Mechanical properties of PA 12 material (top) and uniaxial plastic strain test data [59] (bottom).

Mechanical Properties		Value	
Density		1.01 g/cm${}^{3}$ [66]	
Young’s modulus		1128 MPa [67]	
Poisson’s ratio		0.3 [66]	
Yield strength		22.8 MPa [59]	
Ultimate strength		46 MPa [66]	
**True Stress** **(MPa)**	**True Plastic** **Strain (mm/mm)**	**True Stress** **(MPa)**	**True Plastic** **Strain (mm/mm)**
22.8	0	40.9	0.045
27.6	0.007	47.6	0.085
31.3	0.014	51.0	0.129
35.6	0.025	51.7	0.150

**Table 3:** Mesh settings for finite element analysis.

Settings	Element Size	Relevance Center	Smoothing	Transition	Span Angle Center
	0.5 mm	Fine	High	Slow	Fine

**Table 4:** Comparison between FEA-estimated stiffness and experimental stiffness of kirigami samples with different notch shapes and areas.

Notch Shape	Notch Area (mm^2^)	FEA Estimated Stiffness (N/mm)	Experimental Stiffness (N/mm)	Error (N/mm)	Error Percentage (%)
Circle	1.77	9.68	9.21	−0.47	−4.83
	3.14	8.39	8.23	−0.17	−1.97
	4.91	7.18	7.71	0.53	7.40
	7.07	6.01	6.56	0.55	9.21
	9.62	4.69	5.19	0.49	10.49
Octagon	1.77	9.50	9.25	−0.25	−2.65
	3.14	8.15	7.87	−0.28	−3.43
	4.91	6.88	7.03	0.15	2.18
	7.07	5.69	6.12	0.43	7.63
	9.62	4.43	4.97	0.53	12.06
Heptagon	1.77	9.63	10.00	0.37	3.87
	3.14	8.07	8.11	0.04	0.52
	4.91	6.77	6.52	−0.25	−3.69
	7.07	5.56	5.92	0.36	6.47
	9.62	4.27	4.85	0.58	13.68
Hexagon	1.77	9.31	9.21	−0.11	−1.14
	3.14	7.89	7.81	−0.08	−1.03
	4.91	6.62	6.58	−0.04	−0.63
	7.07	5.41	6.11	0.70	12.91
	9.62	4.15	4.80	0.65	15.60
Pentagon	1.77	9.12	8.41	−0.71	−7.81
	3.14	7.68	8.03	0.35	4.61
	4.91	6.36	6.22	−0.14	−2.18
	7.07	5.16	5.60	0.44	8.48
	9.62	3.96	4.65	0.69	17.41
Square	1.77	8.71	9.15	0.44	5.03
	3.14	7.18	7.74	0.56	7.81
	4.91	5.78	6.23	0.45	7.80
	7.07	4.46	4.80	0.35	7.77
	9.62	3.20	3.84	0.64	20.01
Triangle	1.77	7.18	6.64	−0.54	−7.46
	3.14	5.42	5.54	0.12	2.23
	4.91	3.91	4.15	0.25	6.30
	7.07	2.68	3.07	0.39	14.56
	9.62	1.68	1.96	0.28	16.49

## Data Availability

Not applicable.

## Abbreviations

The following abbreviations are used in this manuscript:

FEA	finite element analysis
MJF	multi-jet fusion
FFF	fused filament fabrication
PBF	powder bed fusion
SLS	selective laser sintering
TPU	Thermoplastic Polyurethane
PA	polyamide
CAD	computer-aided design
DAQ	data acquisition
ISO	International Organization for Standardization
ASTM	American Society for Testing and Materials

## Appendix A. Additional Finite Element Analysis Data

**Table A1:** Computational yielding points of kirigami-inspired samples in finite element analysis.

		Notch Area (mm_2_)	1.77	3.14	4.91	7.07	9.62
	Yield Deformation $\epsilon_{\mathrm{Yield}}$ (mm)						
Notch Shape							
Circle			2.04	2.49	2.81	3.07	3.50
Octagon			2.01	2.33	2.49	2.75	3.22
Heptagon			1.99	2.32	**2.41**	2.80	**2.94**
Hexagon			**1.98**	**2.09**	2.54	**2.60**	3.18
Pentagon			2.87	3.38	3.74	4.14	4.50
Square			3.18	3.69	4.36	5.38	5.60
Triangle			3.56	4.33	5.52	5.29	6.75

**Table A2:** Yield energy of kirigami-inspired samples when stretched to the value indicated in Table A1 with bold type for corresponding notch areas.

Notch Shape	Strain Energy U (mJ) (1.77 mm^2^)	Strain Energy U (mJ) (3.14 mm^2^)	Strain Energy U (mJ)(4.91 mm^2^)	Strain Energy U (mJ) (7.07 mm^2^)	Strain Energy U (mJ) (9.62 mm^2^)
Circle	18.95	18.31	20.82	20.29	20.29
Octagon	18.60	17.80	19.97	19.23	19.16
Heptagon	18.45	17.62	19.65	18.80	18.46
Hexagon	18.24	17.23	19.23	18.30	17.95
Pentagon	17.82	16.72	18.43	17.43	17.11
Square	16.99	15.64	16.75	15.04	13.82
Triangle	14.00	11.80	11.33	9.06	7.30

## Appendix C. Finite Element Analysis Results of Slit Height-Reduced Kirigami Samples

**Table A3:** Computational yielding points and estimated stiffness of kirigami-inspired samples with octagonal, heptagonal, and hexagonal shape notches in finite element analysis.

Notch Shape	Yield Deformation $\epsilon_{\mathrm{Yield}}$ (mm)	FEA Estimated Stiffness (N/mm)
Octagonal	1.69	12.24
Heptagonal	1.77	12.17
Hexagonal	1.89	12.04
